# Increased incidence of sudden sensorineural hearing loss in patients with rheumatoid arthritis: a nationwide propensity-matched cohort study

**DOI:** 10.1016/j.clinsp.2026.100990

**Published:** 2026-05-14

**Authors:** Ming-Jenn Chen, Yu-Cheng Lee, Ming-Yao Chen, Chi-Hsiang Chung, Wu-Chien Chien, Ding-Yen Lin, Yung-Fu Wu, Kuen-Haur Lee

**Affiliations:** a Department of Surgery, Chi Mei Medical Center, Tainan, Taiwan; b Department of Sports Management, College of Leisure and Recreation Management, Chia Nan University of Pharmacy and Science, Tainan, Taiwan; c Graduate Institute of Medical Sciences, College of Medicine, Taipei Medical University, Taiwan; d Division of Gastroenterology and Hepatology, Department of Internal Medicine, College of Medicine, Taipei Medical University, Taiwan; e Division of Gastroenterology and Hepatology, Department of Internal Medicine, Taipei Medical University Shuang Ho Hospital, Ministry of Health and Welfare, Taiwan; f Department of Medical Research, Tri-Service General Hospital, National Defense Medical Center, Taiwan; g Department of Biotechnology and Bioindustry Sciences, College of Bioscience and Biotechnology, National Cheng Kung University, Taiwan; h Department of Pharmacology, College of Medicine, National Cheng Kung University, Taiwan; i Program for Cancer Molecular Biology and Drug Discovery, College of Medical Science and Technology, Taipei Medical University-Shuangho Campus, Taiwan; j Graduate Institute of Cancer Molecular Biology and Drug Discovery, College of Medical Science and Technology, Taipei Medical University-Shuangho Campus, New Taiwan, Taiwan; k TMU Research Center for Digestive Medicine, Taipei Medical University, Taiwan; l Cancer Center, Wan Fang Hospital, Taipei Medical University, Taiwan; m TMU Research Center of Cancer Translational Medicine, Taipei Medical University, , Taiwan

**Keywords:** Sudden sensorineural hearing loss, Rheumatoid arthritis, Autoimmune disease, Cohort study, National health insurance database, Hearing loss screening

## Abstract

**Objectives:**

To determine whether Rheumatoid Arthritis (RA) is associated with an increased risk of incident Sudden Sensorineural Hearing Loss (SSNHL).

**Methods:**

The authors conducted a nationwide, propensity-score-matched cohort study using Taiwan’s Longitudinal Health Insurance Database (2000–2015). Adults with incident RA (n = 45,425) were matched 1:4 to those without RA controls (n = 181,700). The primary outcome was incident SSNHL (ICD-9-CM 389.10). Time-to-event analyses used Kaplan-Meier curves with log-rank tests and multivariable Cox regression. Landmark sensitivity analyses excluded SSNHL events within the first 1- and, separately, 5-years of follow-up.

**Results:**

SSNHL occurred in 169/45,425 (0.37%) RA patients and 462/181,700 (0.25%) controls. RA was associated with a higher SSNHL risk (adjusted hazard ratio 2.695, 95% CI 2.248–3.231; p < 0.001). Kaplan-Meier and log-rank tests showed greater cumulative incidence in the RA cohort (p < 0.001). In landmark sensitivity analyses excluding events within the first 1- and 5-years, the association remained significant (Year 1: 34 vs. 45, p = 0.007; Year 5: 105 vs. 172, p < 0.001), indicating that the excess risk was not driven by very early events.

**Conclusions:**

The present study revealed a high frequency of SSNHL in patients with RA, which highlights the importance of heightened clinical vigilance and prompt audiologic evaluation for hearing-related symptoms in this population.

## Highlights

• RA patients show significantly increased SSNHL risk.
• Large-scale nationwide cohort strengthens validity.
• Propensity matching reduces confounding bias.
• RA increases SSNHL risk by approximately 2.7-fold.
• Consistent findings across stratified and sensitivity analyses.

## Introduction

Sudden Sensorineural Hearing Loss (SSNHL), previously and still more commonly known as sudden deafness, is a type of hearing loss characterized by a rapid perception of hearing loss over a brief period, usually in one ear.^1^ The most widely used definition of SSNHL is at least 30 dB of loss at three consecutive frequencies, and this condition represents otologic urgency that requires immediate evaluation and treatment to optimize hearing recovery within 72 h.^2^ SSNHL has an annual incidence of approximately 5–20 per 100,000 patients, and it is slightly higher in those aged 40–60 years.^3^ SSNHL is characterized by sudden, often unilateral hearing loss. This might be associated with tinnitus (a sensation of ringing in the ear), aural fullness, and, in some cases, vertigo.^4^ Some may have mild hearing loss, and others may have a profound level of hearing loss. Although there have been many research efforts, the precise mechanisms leading to SSNHL remain largely unknown. SSNHL is a complex phenomenon with multifactorial causes, including idiopathic and secondary factors such as infections, vascular events, ototoxic medications, and autoimmune disorders, including Cogan syndrome.

Autoimmune diseases have been increasingly suggested to be potential contributors to the occurrence and development of SSNHL.^5^ Presently, the evidence for this view is incompletely documented. SSNHL was reported in some case reports and clinical trials to be connected with systemic autoimmune disorders or symptoms, such as autoimmune hepatitis, sinusitis, headaches, sympathetic hyperalgesia, edema syndrome, Cogan's syndrome, Systemic Lupus Erythematosus (SLE), Multiple Sclerosis (MS), Crohn's disease, and Rheumatoid Arthritis (RA).^6^ RA is an ongoing systemic autoimmune disease characterized by continuous inflammation of the synovial membranes of the joints, which may cause joint destruction, loss of function, and deformity, as well as long-term discomfort.^7^ Moreover, RA may cause other symptoms and multi-system, multi-organ, and multi-tissue complications, including cardiocerebrovascular, pulmonary, and neurological complications.^8^ Emerging evidence suggests that RA may also be linked to auditory dysfunction, specifically SSNHL. Hearing loss is speculated to arise from autoimmune mechanisms acting on the cochlea. It is possible that cochlear damage in RA may be caused by inflammatory processes that subsequently lead to hearing impairments. Immune-mediated inner ear injury has been proposed to involve small-vessel vasculitis, immune complex deposition, and microvascular compromise affecting the cochlear circulation.^9^ In addition, circulating autoantibodies and autoreactive immune responses targeting inner ear antigens may disrupt cochlear homeostasis and precipitate acute sensorineural hearing loss, particularly in systemic autoimmune conditions characterized by chronic inflammation, such as rheumatoid arthritis.^10^

Despite increasing recognition of autoimmune contributions to SSNHL, evidence from large, population-based cohorts remains limited. The authors therefore conducted a nationwide, propensity-score-matched cohort study using Taiwan’s National Health Insurance Research Database (NHIRD) to test whether RA is associated with an increased risk of incident SSNHL. The primary outcome was incident SSNHL, and risk was evaluated using Kaplan-Meier/log-rank and multivariable Cox regression, with landmark sensitivity analyses to address potential protopathic bias.

## Materials and methods

### Data sources

This study is based on information from a population examined from the NHIRD in Taiwan. The NHI program began in 1995 and now includes >99% of the population, serving about 23 million people.^11^ The NHIRD uses the International Classification of Diseases, 9th Revision, Clinical Modification (ICD-9-CM), which is utilized to document diagnoses.^12^ An analysis was conducted using a portion of the database known as the NHIRD to investigate the link between sensorineural hearing loss and RA occurrence. The NHIRD comprises information gathered from 1 million individuals chosen at random from the NHI enrollee population in 2000. For the present study, datasets were used to match patients from data collected between January 1, 2000, and December 31, 2015. Corresponding diseases of interest were diagnosed using ICD-9-CM codes. Statement that all data are available within the text.

### Study design and sample population

**Fig. 1:**
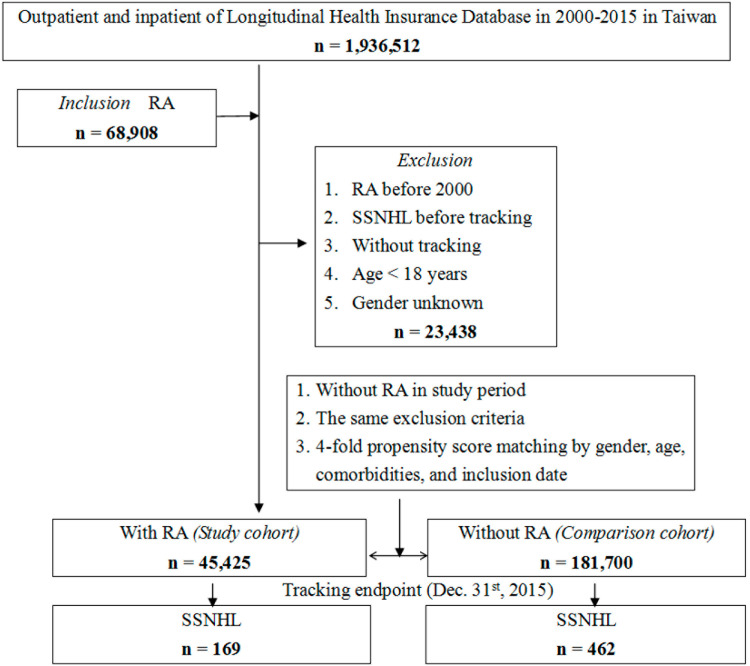
**Flowchart of the study**. Controls were selected via 1:4 propensity score matching on age, sex, index year (inclusion date), and baseline comorbidities.

This retrospective population-based cohort study selected the study population as depicted in Fig. 1. A flowchart of the study sample selection is similar to the previous report.^13^ The study selected patients diagnosed with RA using ICD-9-CM code 714.0, documented in the NHIRD between January 1, 2000, and December 31, 2015. This study adhered to STROBE guidelines. Exclusion criteria included (i) Patients who had RA before the index date; (ii) Patients with SSNHL before the tracking period; (iii) Patients younger than 18-years; and (iv) Those with an unspecified gender. In total, 181,700 patients without RA were randomly selected as the comparison cohort, and the authors used nearest-neighbor propensity score matching without replacement in a 1:4 ratio (RA: non-RA) with a caliper width of 0.2 standard deviations of the logit of the propensity score. The propensity score was estimated via logistic regression including age, sex, index year (inclusion date), and baseline comorbidities (as defined in Supplementary Table S1: DM, HTN, depression, anxiety, CKD, hyperlipidemia, thyrotoxicosis, septicemia, pneumonia, CLD, injury, tumor, neurofibromatosis). Follow-up began at the index date, defined as the first qualifying RA diagnosis for cases and the matched index year anchor for controls, and time-to-event analyses were conducted using Kaplan-Meier curves, log-rank tests, and multivariable Cox regression.

### Outcome measures

The authors followed up all study patients from the index date until the onset of SSNHL or the end of 2015. ICD-9-CM definitions for SSNHL (389.10), RA (714.0), and all comorbidities are summarized in Supplementary Table S1.

### Potential confounders

The authors considered various confounding factors such as sex, age or age group, geographic location in Taiwan, urbanization level, insurance premium, season, and level of care. Individuals with and those without comorbidities mentioned in Table 1 before or on the index date were organized for comparison.

**Table 1:** Characteristics of the study population at the baseline.

**RA Variable**	**Total**		**With**		**Without**		**p-value**
	**n**	**%**	**n**	**%**	**n**	**%**	
**Total**	227,125		45,425	20.00	181,700	80.00	
**Gender**							0.999
Male	61,005	26.86	12,201	26.86	48,804	26.86	
Female	166,120	73.14	33,224	73.14	132,896	73.14	
**Age (years)**	58.87 ± 16.05		58.77 ± 15.72		58.89 ± 16.13		0.154
**Age groups (years)**							0.999
18–44	34,370	15.13	6874	15.13	27,496	15.13	
45–64	101,060	44.50	20,212	44.50	80,848	44.50	
≥ 65	91,695	40.37	18,339	40.37	73,356	40.37	
**Insurance premium (NT$)**							0.069
< 18,000	165,097	72.69	33,201	73.09	131,896	72.59	
18,000–34,999	39,920	17.58	7825	17.23	32,095	17.66	
≥ 35,000	22,108	9.73	4399	9.68	17,709	9.75	
**DM**							0.658
Without	175,237	77.15	35,012	77.08	140,225	77.17	
With	51,888	22.85	10,413	22.92	41,475	22.83	
**HTN**							0.270
Without	182,423	80.32	36,401	80.13	146,022	80.36	
With	44,702	19.68	9024	19.87	35,678	19.64	
**Depression**							0.971
Without	189,463	83.42	37,890	83.41	151,573	83.42	
With	37,662	16.58	7535	16.59	30,127	16.58	
**Anxiety**							0.728
Without	191,246	84.20	38,225	84.15	153,021	84.22	
With	35,879	15.80	7200	15.85	28,679	15.78	
**CKD**							0.990
Without	195,609	86.12	39,121	86.12	156,488	86.12	
With	31,516	13.88	6304	13.88	25,212	13.88	
**Hyperlipidemia**							0.842
Without	200,499	88.28	40,112	88.30	160,387	88.27	
With	26,626	11.72	5313	11.70	21,313	11.73	
**Thyrotoxicosis**							0.779
Without	221,217	97.40	44,235	97.38	176,982	97.40	
With	5908	2.60	1190	2.62	4718	2.60	
**Septicemia**							0.870
Without	222,300	97.88	44,465	97.89	177,835	97.87	
With	4825	2.12	960	2.11	3865	2.13	
**Pneumonia**							0.921
Without	198,906	87.58	39,775	87.56	159,131	87.58	
With	28,219	12.42	5650	12.44	22,569	12.42	
**CLD**							0.896
Without	200,565	88.31	40,121	88.32	160,444	88.30	
With	26,560	11.69	5304	11.68	21,256	11.70	
**Injury**							0.552
Without	194,060	85.44	38,852	85.53	155,208	85.42	
With	33,065	14.56	6573	14.47	26,492	14.58	
**Tumor**							0.912
Without	200,594	88.32	40,112	88.30	160,482	88.32	
With	26,531	11.68	5313	11.70	21,218	11.68	
**Neurofibromatosis**							0.520
Without	225,107	99.11	45,010	99.09	180,097	99.12	
With	2018	0.89	415	0.91	1603	0.88	
**CCI_R**	1.00 ± 1.03		1.01 ± 1.04		1.00 ± 1.03		0.065
**Season**							0.999
Spring (Mar. – May)	56,010	24.66	11,202	24.66	44,808	24.66	
Summer (June – Aug.)	61,750	27.19	12,350	27.19	49,400	27.19	
Autumn (Sept. – Nov.)	56,200	24.74	11,240	24.74	44,960	24.74	
Winter (Dec. – Feb.)	53,165	23.41	10,633	23.41	42,532	23.41	
**Location**							0.006^a^
Northern Taiwan	66,381	29.23	13,256	29.18	53,125	29.24	
Central Taiwan	58,848	25.91	11,782	25.94	47,066	25.90	
Southern Taiwan	56,195	24.74	11,303	24.88	44,892	24.71	
Eastern Taiwan	34,824	15.33	6789	14.95	28,035	15.43	
Outlying islands	10,877	4.79	2295	5.05	8582	4.72	
**Urbanization level**							<0.001^b^
1 (highest)	64,888	28.57	12,892	28.38	51,996	28.62	
2	71,395	31.43	14,297	31.47	57,098	31.42	
3	42,372	18.66	8922	19.64	33,450	18.41	
4 (lowest)	48,470	21.34	9314	20.50	39,156	21.55	<0.001^b^
**Level of care**							
Hospital center	59,110	26.03	12,015	26.45	47,095	25.92	
Regional hospital	92,091	40.55	18,970	41.76	73,121	40.24	
Local hospital	75,924	33.43	14,440	31.79	61,484	33.84	

p, Chi-Squared/Fisher’s exact test on categorical variables and *t*-test on continuous variables. RA, Rheumatoid Arthritis; DM, Diabetes Mellitus; NT$, New Taiwan dollar; HTN, Hypertension; CKD, Chronic Kidney Disease; CLD, Chronic Lung Disease; CCI_R, Revised Charlson Comorbidity Index. ICD-9-CM codes and definitions are shown in Table S1.

a p < 0.01.

b p < 0.001.

### Data analysis

Chi-squared and *t*-tests were respectively used for categorical and continuous types of variables. For comparisons of the two groups with categorical variables, both groups utilized Fisher’s exact test. The Relative Risk (RR)/Hazard Ratio (HR) with the 95% CI was computed by utilizing a multivariate Cox proportional hazards regression analysis. To address potential protopathic bias, the authors performed landmark analyses by excluding SSNHL events occurring within the first 1-year and, separately, within the first 5-years of follow-up. The authors then reapplied Kaplan-Meier and log-rank comparisons between RA and non-RA cohorts on the restricted risk sets. About the multiple comparison correction, the prespecified primary inference was the association between RA and incident SSNHL; this effect was tested at a two-sided α = 0.05 without multiplicity adjustment. For secondary inferences, the authors controlled the false discovery rate at 5% (Benjamini-Hochberg) and present raw p-values for transparency. Specifically, secondary analyses included subgroup and stratified Cox models by age, sex, comorbidities, geographic region, and level of care. Results with p < 0.001 remained significant under Bonferroni correction. The significance level was two-tailed at 0.05. All statistical analyses were performed using SPSS software (vers. 22.0, IBM, Chicago, IL, USA). This research was registered with the Policy of the World Medical Association (WMA) on ethical issues in medicine (Helsinki statement).

### Ethics statement

The study protocol was approved by the Institutional Review Board (IRB approval n°: E202416032). Informed consent was waived because the identification information in Taiwan’s NHIRD is encrypted to ensure privacy.

## Results

### Demographic characteristics at the baseline and endpoint

**Fig. 2:**
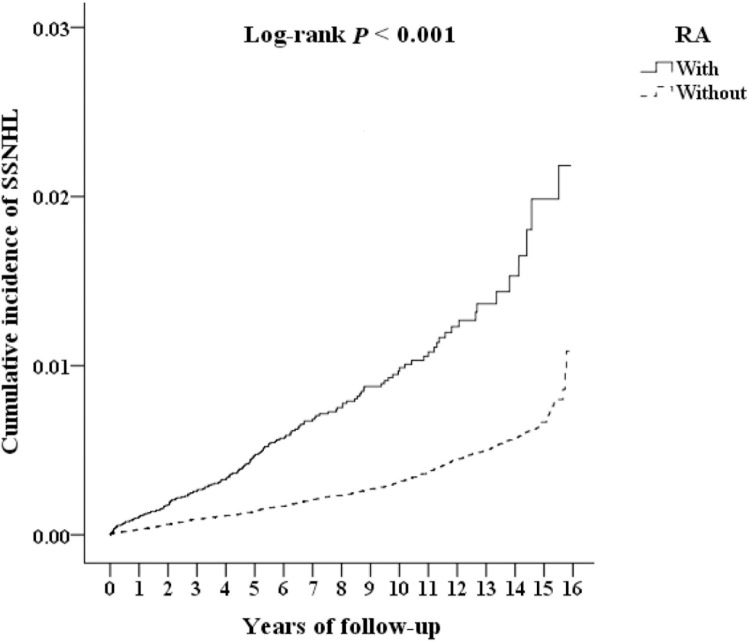
Kaplan-Meier analysis for the cumulative incidence of Sudden Sensorineural Hearing Loss (SSNHL) aged 18-years and over stratified by patients with Rheumatoid Arthritis (RA) (solid line) and without RA (dotted line) according to a log-rank test.

In 2000–2015 records from the NHIRD 2005, of the 1949,101 patients with outpatient or inpatient records, the authors identified patients who met the inclusion criteria among the 45,425 patients diagnosed with RA in 2000–2015 and found 169 patients with both RA and SSNHL and also included a control group of 181,700 patients without RA, among which 462 individuals had an SSNHL diagnosis (Fig. 1). Baseline and Endpoint Characteristics (Table 1, Table 2). At baseline (Table 1), the RA and non-RA cohorts differed in geographic location (p = 0.006) and urbanization level (p < 0.001), while sex and age were balanced by design; there were no statistically significant differences found between the two cohorts in percentages of patients with Diabetes Mellitus (DM), Hypertension (HTN), depression, anxiety, Chronic Kidney Disease (CKD), hyperlipidemia, thyrotoxicosis, septicemia, pneumonia, Chronic Lung Disease (CLD), injury, tumor, neurofibromatosis, or the Charlson Comorbidity Index, Revised (CCI_R) (all p > 0.05), (Table 1, Supplementary Table S1). By the end of follow-up (Table 2), 169/45,425 (0.37%) patients with RA and 462/181,700 (0.25%) patients without RA had developed SSNHL (p < 0.001), and the cohorts differed in age distribution, place of residence, and level of care (all p < 0.001), whereas the above comorbidities and CCI_R remained not significantly different between groups (all p > 0.05; Table 2). Median follow-up was 7.26-years (RA) and 7.75-years (controls) with similar ranges (Supplementary Table S2‒1). Among SSNHL cases, the median time-to-event was 4.01-years (RA) and 6.94-years (controls) (Supplementary Table S2‒2). Consistent with these table-level findings, Kaplan-Meier curves and log-rank tests demonstrated a higher cumulative incidence of SSNHL in the RA cohort (p < 0.001; Fig. 2; Supplementary Tables S2‒1, S2‒2), indicating that while contextual factors (location/urbanization and, at endpoint, age/residence/care level) differed between cohorts, medical comorbidity profiles were comparable, and the excess SSNHL risk in RA was observed across follow-up after accounting for these contextual differences.

**Table 2:** Characteristics of study population at the endpoint.

**RA**	**Total**		**With**		**Without**		**p-value**
**Variables**	**n**	**%**	**n**	**%**	**n**	**%**	
**Total**	227,125		45,425	20.00	181,700	80.00	
**SSNHL**							<0.001^a^
Without	226,494	99.72	45,256	99.63	181,238	99.75	
With	631	0.28	169	0.37	462	0.25	
**Gender**							0.999
Male	61,005	26.86	12,201	26.86	48,804	26.86	
Female	166,120	73.14	33,224	73.14	132,896	73.14	
**Age (years)**	68.44 ± 17.12		67.90 ± 16.25		68.57 ± 17.33		<0.001^a^
**Age groups (years)**							<0.001^a^
18–44	29,201	12.86	6377	14.04	22,824	12.56	
45–64	70,835	31.19	16,849	37.09	53,986	29.71	
≥ 65	127,089	55.96	22,199	48.87	104,890	57.73	
**Insured premium (NT$)**							0.069
< 18,000	165,097	72.69	33,201	73.09	131,896	72.59	
18,000–34,999	39,920	17.58	7825	17.23	32,095	17.66	
≥ 35,000	22,108	9.73	4399	9.68	17,709	9.75	
**DM**							0.172
Without	175,008	77.05	34,892	76.81	140,116	77.11	
With	52,117	22.95	10,533	23.19	41,584	22.89	
**HTN**							0.803
Without	181,600	79.96	36,301	79.91	145,299	79.97	
With	45,525	20.04	9124	20.09	36,401	20.03	
**Depression**							0.111
Without	188,857	83.15	37,885	83.40	150,972	83.09	
With	38,268	16.85	7540	16.60	30,728	16.91	
**Anxiety**							0.148
Without	190,498	83.87	38,201	84.10	152,297	83.82	
With	36,627	16.13	7224	15.90	29,403	16.18	
**CKD**							0.558
Without	195,314	85.99	39,024	85.91	156,290	86.02	
With	31,811	14.01	6401	14.09	25,410	13.98	
**Hyperlipidemia**							0.563
Without	200,303	88.19	40,025	88.11	160,278	88.21	
With	26,822	11.81	5400	11.89	21,422	11.79	
**Thyrotoxicosis**							0.396
Without	220,366	97.02	44,101	97.09	176,265	97.01	
With	6759	2.98	1324	2.91	5435	2.99	
**Septicemia**							0.816
Without	222,147	97.81	44,423	97.79	177,724	97.81	
With	4978	2.19	1002	2.21	3976	2.19	
**Pneumonia**							0.314
Without	198,822	87.54	39,701	87.40	159,121	87.57	
With	28,303	12.46	5724	12.60	22,579	12.43	
**CLD**							0.767
Without	200,266	88.17	40,035	88.13	160,231	88.18	
With	26,859	11.83	5390	11.87	21,469	11.82	
**Injury**							0.831
Without	193,988	85.41	38,812	85.44	155,176	85.40	
With	33,137	14.59	6613	14.56	26,524	14.60	
**Tumor**							0.987
Without	200,485	88.27	40,098	88.27	160,387	88.27	
With	26,640	11.73	5327	11.73	21,313	11.73	
**Neurofibromatosis**							0.598
Without	225,078	99.10	45,006	99.08	180,072	99.10	
With	2047	0.90	419	0.92	1628	0.90	
**CCI_R**	1.01 ± 1.02		1.02 ± 1.04		1.01 ± 1.01		0.061
**Season**							0.064
Spring	55,975	24.65	11,267	24.80	44,708	24.61	
Summer	60,269	26.54	12,010	26.44	48,259	26.56	
Autumn	57,147	25.16	11,246	24.76	45,901	25.26	
Winter	53,734	23.66	10,902	24.00	42,832	23.57	
**Location**							<0.001^a^
Northern Taiwan	66,381	29.23	13,252	29.17	53,129	29.24	
Central Taiwan	58,496	25.75	11,375	25.04	47,121	25.93	
Southern Taiwan	57,003	25.10	11,202	24.66	45,801	25.21	
Eastern Taiwan	34,567	15.22	6586	14.50	27,981	15.40	
Outlying islands	10,678	4.70	3010	6.63	7668	4.22	
**Urbanization level**							<0.001^a^
1 (highest)	64,410	28.36	12,813	28.21	51,597	28.40	
2	71,290	31.39	14,265	31.40	57,025	31.38	
3	42,444	18.69	8996	19.80	33,448	18.41	
4 (lowest)	48,981	21.57	9351	20.59	39,630	21.81	
**Level of care**							<0.001^a^
Hospital center	59,076	26.01	12,090	26.62	46,986	25.86	
Regional hospital	92,130	40.56	18,965	41.75	73,165	40.27	
Local hospital	75,919	33.43	14,370	31.63	61,549	33.87	

p, Chi-Squared/Fisher’s exact test on categorical variables and *t*-test on continuous variables. RA, Rheumatoid Arthritis; SSNHL, Sudden Sensorineural Hearing Loss; DM, Diabetes Mellitus; HTN, Hypertension; NT$, New Taiwan dollar; CKD, Chronic Kidney Disease; CLD, Chronic Lung Disease; CCI_R, Revised Charlson Comorbidity index.

a p < 0.001.

### Risk factors for developing SSNHL

When adjusted for other variables, an HR of 2.695 (95% CI 2.248–3.231, p < 0.001) was reported during evaluation of RA patients (Table 3). This study demonstrated a great connection between the development of RA and SSNHL (p < 0.001) (Table 3). A significantly greater risk of SSNHL was identified in subjects aged 45–64 years, with DM, HTN, depression, anxiety, CKD, thyrotoxicosis, septicemia, pneumonia, CLD, injury, a tumor, neurofibromatosis, or CCI_R (Table 3). The authors further investigated risk factors for SSNHL using multivariable Cox regression analyses stratified by various demographic and clinical characteristics (Table 4). After stratification by sex, both male and female patients with RA exhibited a significantly increased risk of SSNHL compared with their non-RA counterparts (Male: aHR = 2.711, 95% CI 2.262‒3.250; Female: aHR = 2.689, 95% CI 2.243‒3.224; both p < 0.001). The association remained consistently significant across all other subgroups analyzed, including age, insurance premium, comorbidities, geographic location, season, and level of care (all p < 0.001), indicating that the elevated risk was not confined to any specific subgroup.

**Table 3:** Factors of sudden sensorineural hearing loss (SSNHL) using a Cox regression.

**Variables**	**Crude HR**	**95% CI**	**95% CI**	**p**	**Adjusted HR**	**95% CI**	**95% CI**	**p**
**RA**								
Without	Reference				Reference			
With	3.013	2.515	3.610	<0.001^c^	2.695	2.248	3.231	<0.001^c^
**Gender**								
Male	1.151	0.968	1.368	0.112	1.169	0.983	1.390	0.078
Female	Reference				Reference			
**Age groups (yrs)**								
18‒44	Reference				Reference			
45‒64	1.550	1.184	2.028	0.001^b^	1.483	1.133	1.941	0.004^b^
≥ 65	0.446	0.339	0.588	<0.001^c^	0.454	0.344	0.598	<0.001^c^
**Insured premium (NT$)**								
< 18,000	Reference				Reference			
18,000‒34,999	0.986	0.565	1.782	0.430	0.922	0.551	1.700	0.452
≥ 35,000	0.832	0.486	1.565	0.513	0.813	0.445	1.502	0.533
**DM**								
Without	Reference				Reference			
With	1.989	1.331	2.765	<0.001^c^	1.808	1.224	2.675	<0.001^c^
**HTN**								
Without	Reference				Reference			
With	2.245	1.453	2.865	<0.001^c^	2.020	1.330	2.762	<0.001^c^
**Depression**								
Without	Reference				Reference			
With	1.865	1.201	2.486	<0.001^c^	1.662	1.050	2.299	0.003^b^
**Anxiety**								
Without	Reference				Reference			
With	1.972	1.234	2.535	<0.001^c^	1.782	1.053	2.365	0.001^b^
**CKD**								
Without	Reference				Reference			
With	2.235	1.865	2.993	<0.001^c^	2.020	1.654	2.782	<0.001^c^
**Hyperlipidemia**								
Without	Reference				Reference			
With	1.532	1.024	1.906	0.025^a^	1.237	0.898	1.483	0.201
**Thyrotoxicosis**								
Without	Reference				Reference			
With	3.201	1.250	5.206	<0.001^c^	2.121	1.004	4.504	0.044^a^
**Septicemia**								
Without	Reference				Reference			
With	3.365	1.486	6.672	<0.001^c^	2.174	1.018	3.972	0.033^a^
**Pneumonia**								
Without	Reference				Reference			
With	2.780	1.509	3.010	<0.001^c^	1.986	1.277	2.685	<0.001^c^
**CLD**								
Without	Reference				Reference			
With	2.100	1.452	2.997	<0.001^c^	1.865	1.231	2.604	<0.001^c^
**Injury**								
Without	Reference				Reference			
With	1.702	1.220	2.304	<0.001^c^	1.425	1.033	2.098	0.016^a^
**Tumor**								
Without	Reference				Reference			
With	1.432	1.365	1.572	<0.001^c^	1.201	1.118	1.354	<0.001^c^
**Neurofibromatosis**								
Without	Reference				Reference			
With	15.224	0.124	159.401	0.945	4.297	0.064	92.030	0.972
**CCI_R**	1.035	1.022	1.057	0.027^a^	1.033	1.021	1.048	0.030^a^
**Season**								
Spring	Reference				Reference			
Summer	1.352	0.865	1.772	0.371	1.242	0.752	1.728	0.389
Autumn	0.997	0.578	1.335	0.435	0.986	0.521	1.312	0.486
Winter	0.924	0.535	1.307	0.472	0.911	0.505	1.285	0.499
**Location**								
Northern Taiwan	Reference				**Multicollinearity with urbanization level**			
Middle Taiwan	0.950	0.511	1.425	0.426	**Multicollinearity with urbanization level**			
Southern Taiwan	0.972	0.523	1.477	0.488	**Multicollinearity with urbanization level**			
Eastern Taiwan	0.881	0.489	1.232	0.523	**Multicollinearity with urbanization level**			
Outlets islands	0.572	0.156	0.986	0.029^a^	**Multicollinearity with urbanization level**			
**Urbanization level**								
1 (The highest)	2.246	1.486	2.972	<0.001^c^	2.018	1.202	2.725	<0.001^c^
2	1.572	1.042	1.883	0.006^b^	1.435	0.953	1.625	0.072
3	1.131	0.589	1.401	0.482	1.020	0.488	1.325	0.530
4 (The lowest)	Reference				Reference			
**Level of care**								
Hospital center	2.567	2.140	3.386	<0.001^c^	1.786	1.335	2.011	<0.001^c^
Regional hospital	2.186	1.782	2.997	<0.001^c^	1.452	1.121	1.706	<0.001^c^
Local hospital	Reference				Reference			

HR, Hazard Ratio; CI, Confidence Interval; DM, Diabetes Mellitus; HTN, Hypertension; NT$, New Taiwan dollar; CKD, Chronic Kidney Disease; CLD, Chronic Lung Disease; CCI_R, Revised Charlson Comorbidity Index.

a p < 0.05.

b p < 0.01.

c p < 0.001.

**Table 4:** Factors of sudden sensorineural hearing loss (SSNHL) stratified by variables listed in the table using a Cox regression.

**RA**	**With**			**Without (Reference)**			**With vs. Without (Reference)**			
**Stratified**	**Events**	**PYs**	**Rate (per 10^5^ PYs)**	**Events**	**PYs**	**Rate (per 10^5^ PYs)**	**Adjusted HR**	**95% CI**	**95% CI**	**p**
**Total**	169	414,730.25	40.75	462	1746,137.01	26.46	2.695	2.248	3.231	<0.001^c^
**Gender**										
Male	46	111,395.11	41.29	125	469,006.44	26.65	2.711	2.262	3.250	<0.001^c^
Female	123	303,335.14	40.55	337	1277,130.57	26.39	2.689	2.243	3.224	<0.001^c^
**Age groups (yrs)**										
18‒44	23	58,222.06	39.50	55	219,386.22	25.07	2.757	2.300	3.306	<0.001^c^
45‒64	63	153,831.26	40.75	133	518,805.64	25.64	2.781	2.320	3.335	<0.001^c^
≥ 65	83	202,676.93	41.11	274	1007,945.15	27.18	2.646	2.207	3.172	<0.001^c^
**Insured premium (NT$)**										
< 18,000	129	303,125.22	42.56	339	1267,503.25	26.75	2.784	2.323	3.338	<0.001^c^
18,000‒34,999	30	71,445.08	41.99	82	308,433.17	26.59	2.764	2.305	3.313	<0.001^c^
≥ 35,000	10	40,159.95	24.90	41	170,200.59	24.09	1.809	1.509	2.169	<0.001^c^
**DM**										
Without	127	318,563.69	39.87	355	1346,486.77	26.36	2.646	2.207	3.172	<0.001^c^
With	42	96,166.56	43.67	107	399,650.24	26.77	2.854	2.381	3.422	<0.001^c^
**HTN**										
Without	132	331,428.13	39.83	368	1396,331.76	26.35	2.644	2.206	3.170	<0.001^c^
With	37	83,302.12	44.42	94	349,805.25	26.87	2.892	2.413	3.468	<0.001^c^
**Depression**										
Without	136	346,324.52	39.27	382	1450,871.93	26.33	2.610	2.177	3.129	<0.001^c^
With	33	68,405.73	48.24	80	295,265.08	27.09	3.116	2.599	3.735	<0.001^c^
**Anxiety**										
Without	137	348,778.12	39.28	391	1513,569.57	25.83	2.661	2.219	3.190	<0.001^c^
With	32	65,952.13	48.52	71	232,567.44	30.53	2.781	2.320	3.334	<0.001^c^
**CKD**										
Without	140	356,022.28	39.32	394	1495,906.21	26.34	2.613	2.179	3.132	<0.001^c^
With	29	58,707.97	49.40	68	250,230.80	27.17	3.181	2.653	3.813	<0.001^c^
**Hyperlipidemia**										
Without	148	365,426.95	40.50	408	1540,835.16	26.48	2.676	2.233	3.209	<0.001^c^
With	21	49,303.30	42.59	54	205,301.85	26.30	2.834	2.364	3.397	<0.001^c^
**Thyrotoxicosis**										
Without	163	402,641.48	40.48	448	1693,906.61	26.45	2.678	2.234	3.211	<0.001^c^
With	6	12,088.77	49.63	14	52,230.40	26.80	3.240	2.703	3.885	<0.001^c^
**Septicemia**										
Without	164	405,582.00	40.44	451	1707,834.00	26.41	2.679	2.235	3.212	<0.001^c^
With	5	9148.25	54.66	11	38,303.01	28.72	3.330	2.778	3.993	<0.001^c^
**Pneumonia**										
Without	148	362,467.28	40.75	405	1529,152.82	26.46	2.695	2.248	3.231	<0.001^c^
With	21	52,262.97	40.75	57	216,984.19	26.46	2.695	2.248	3.231	<0.001^c^
**CLD**										
Without	146	365,519.59	39.94	406	1539,819.06	26.37	2.651	2.211	3.178	<0.001^c^
With	23	49,210.66	46.74	56	206,317.95	27.14	3.013	2.513	3.612	<0.001^c^
**Injury**										
Without	142	353,750.90	40.14	394	1491,280.30	26.42	2.659	2.218	3.187	<0.001^c^
With	27	60,979.35	44.28	68	254,856.71	26.68	2.904	2.422	3.481	<0.001^c^
**Tumor**										
Without	147	366,074.25	40.16	407	1541,319.66	26.41	2.661	2.220	3.190	<0.001^c^
With	22	48,656.00	45.22	55	204,817.35	26.85	2.946	2.458	3.532	<0.001^c^
**Neurofibromatosis**										
Without	167	409,887.59	40.74	459	1734,901.90	26.46	2.695	2.248	3.231	<0.001^c^
With	2	4842.66	41.30	3	11,235.11	26.70	2.706	2.258	3.245	<0.001^c^
**Season**										
Spring	42	102,897.23	40.82	113	429,648.23	26.30	2.716	2.265	3.256	<0.001^c^
Summer	46	109,651.03	41.95	124	463,499.78	26.75	2.744	2.289	3.290	<0.001^c^
Autumn	42	102,675.98	40.91	109	411,108.13	26.51	2.700	2.252	3.237	<0.001^c^
Winter	39	99,506.01	39.19	116	441,880.87	26.25	2.613	2.179	3.132	<0.001^c^
**Location**										
1 (The highest)	50	116,986.29	42.74	133	495,847.14	26.82	2.788	2.326	3.343	<0.001^c^
2	53	130,239.45	40.69	145	548,010.25	26.46	2.691	2.245	3.227	<0.001^c^
3	33	82,133.84	40.18	85	321,435.84	26.44	2.659	2.218	3.187	<0.001^c^
4 (The lowest)	33	85,370.67	38.65	99	380,843.78	25.99	2.602	2.170	3.120	<0.001^c^
**Level of care**										
Hospital center	53	110,381.70	48.02	123	451,535.49	27.24	3.084	2.573	3.698	<0.001^c^
Regional hospital	69	173,150.45	39.85	187	703,115.62	26.60	2.622	2.187	3.143	<0.001^c^
Local hospital	47	131,198.10	35.82	152	591,485.90	25.70	2.439	2.035	2.924	<0.001^c^

PYs, Person-Years; RA, Rheumatoid Arthritis; CI, Confidence Interval; DM, Diabetes Mellitus; HTN, Hypertension; NT$, New Taiwan dollar; CKD, Chronic Kidney Disease; CLD, Chronic Lung Disease; CCI_R, Revised Charlson Comorbidity Index.

^a^ p < 0.05.

^b^ p < 0.01.

c p < 0.001.

### Sensitivity test

A sensitivity analysis addressing potential protopathic bias was performed using landmark (exclusion) analyses with log-rank tests. After excluding SSNHL events within the first 1-year and, separately, within the first 5-years, the RA cohort continued to show a significantly higher cumulative incidence than the non-RA cohort. By Year 1, events were 34 (RA) vs. 45 (non-RA) (p = 0.007); by Year 5, 105 vs. 172 (p < 0.001), with significance persisting thereafter (all p ≤ 0.034 through Year 16), indicating that the excess risk is not driven solely by very early events and that curve separation remains evident beyond Year 5 (Table 5). Table 5 summarizes cumulative events and log-rank p-values annually from Year 1 to Year 16, and Supplementary Tables 2‒2 show median time-to-SSNHL of 4.01-years in RA vs. 6.94-years in non-RA.

**Table 5:** Sensitivity of sudden sensorineural hearing loss (SSNHL) to rheumatoid arthritis (RA) using a Cox regression.

**RA**	**With (n=45,425)**	**Without (n=181,700)**	**Log-rank**
**In the tracking of × year(s)**	**Numbers of SSNHL**		**p**
1	34	45	0.007^b^
2	53	85	0.023^a^
3	72	120	0.026^a^
4	84	145	0.034^a^
5	105	172	<0.001^c^
6	118	200	<0.001^c^
7	130	234	<0.001^c^
8	136	256	<0.001^c^
9	145	283	<0.001^c^
10	151	311	<0.001^c^
11	155	342	<0.001^c^
12	160	383	<0.001^c^
13	163	408	<0.001^c^
14	165	431	<0.001^c^
15	168	449	<0.001^c^
16	169	462	<0.001^c^

a p < 0.05.

b p < 0.01.

c p < 0.001.

## Discussion

In this study, the authors sought to investigate the relationship between SSNHL and RA using the NHIRD to analyze a large Taiwanese population-based cohort study. Evidence suggests that patients with RA are at a higher risk of developing SSNHL compared to the non-RA population. The current finding indeed supports the increasing relevance of autoimmune diseases in hearing dysfunction.^14^, ^15^, ^16^, ^17^, ^18^ The present study revealed a high frequency of SSNHL in patients with RA, which highlights the importance of heightened clinical vigilance and prompt audiologic evaluation for hearing-related symptoms in this population. Since there is inflammatory fibrosis across multiple systems in patients with RA, it is reasonable to hypothesize that immune-mediated damage in the presence of RA goes beyond the joints to the cochlea or auditory pathways, resulting in SSNHL.

This investigation's finding concerning the reason for inflammatory and autoimmune conditions is consistent with the finding of the SSNHL incidence in the RA cohort being higher than that of the non-RA cohort. Even after adjusting for comorbidities such as DM and CKD, RA continued to be an independent factor for SSNHL. This indeed strengthens the premise that immune-driven events are contributors to the etiology of sudden hearing impairment. The RA immune response, which commonly targets the joints, may also attack the inner ear structures, causing cochlear inflammation, microvascular damage, or other pathophysiological processes that may result in sudden deafness.^19^ This linkage is reminiscent of other autoimmune diseases, such as Cogan's syndrome and SLE, that are associated with hearing loss and also involve inner ear structures.^20^, ^21^, ^22^ Mechanistically, several immune-mediated pathways plausibly connect RA with acute cochlear injury: Autoimmune Inner-Ear Disease (AIED) describes antibody- or T-cell-mediated injury to targets such as the stria vascularis and spiral ligament/endolymphatic sac, supporting a direct autoimmune otopathy in susceptible hosts,^20^ while temporal bone histopathology in systemic autoimmunity (such as SLE) demonstrates vasculitis, immune-complex deposition, and hair-cell loss, providing tissue-level evidence of cochlear damage.^21^ In parallel, prothrombotic autoimmunity may impair cochlear microcirculation: antiphospholipid antibodies have been linked to AIED and sudden hearing loss, and experimental work ties sudden deafness to immune-mediated systemic vasculitis.^15^^,^^22^ Additional reports implicate melanocyte- or endothelial-directed autoimmunity in cochlear dysfunction and immune-related otologic adverse events, reinforcing a direct immunologic mechanism beyond nonspecific inflammation.^14^^,^^16^ Notably, vascular and immune pathways likely interact: conventional vascular risks are associated with small-vessel disease in idiopathic SSNHL, yet the RA-SSNHL association persisted after covariate adjustment and in landmark analyses, suggesting an added autoimmune contribution in RA.^23^ Recent metabolomic profiling studies in inflammatory arthritis further support systemic immune-metabolic dysregulation as a contributor to extra-articular manifestations.^24^

In terms of prior evidence, research on RA and auditory dysfunction has been dominated by small clinical series, case reports, and reviews, often using audiometric thresholds rather than incident SSNHL as outcomes and yielding mixed associations.^18^^,^^19^ The AIED literature offers biologic plausibility but is not RA-specific and generally lacks population-level estimates.^14^^,^^20^ Studies in other systemic autoimmune diseases (such as SLE) document inner-ear involvement histologically and clinically,^21^ and antiphospholipid antibodies/vasculitis have been linked to sudden hearing loss in experimental and clinical contexts.^22^ Meanwhile, vascular risk correlates with idiopathic SSNHL in the general population,^23^ but prior work rarely separates autoimmune from vascular pathways or adjusts comprehensively for comorbidities. Against this backdrop, the nationwide propensity-score-matched cohort quantifies the RA-SSNHL association using Cox time-to-event analysis with landmark sensitivity analyses showing that the effect persists beyond early follow-up. These design features ‒ large sample size, population coverage, incident outcome definition (ICD-9-CM 389.10), comprehensive covariate adjustment, and robustness checks ‒ yield a more definitive, population-level estimate (Ahr = 2.695, 95% CI 2.248–3.231) and situate RA within an immune-mediated pathway to SSNHL that is not explained by comorbidity imbalance. Clinically, these converging lines of evidence support heightened audiologic vigilance in RA and timely anti-inflammatory management when SSNHL occurs, consistent with an immune-mediated pathogenesis.

The qualitative analysis further noted that SSNHL was more common in those aged 45–64 years, in men, and in those with other underlying diseases such as DM and HTN. This indicates that the aging process and systemic inflammation, accompanied by comorbid factors, may increase the odds of developing SSNHL among RA patients. It was previously shown that such factors as HTN and DM, which cause microvascular pathology of the cochlea, are linked to hearing loss due to vascular impairment.^23^ On the other hand, the marked rise in SSNHL among RA patients in the absence of these traditional risk factors points towards the involvement of autoimmune mechanisms that are not vascular. Thus, audiometric tests should become part of comprehensive care for RA patients over 45-years of age and in those with other systemic diseases.

The results of this study have significant ramifications for managing and treating RA. It remains unclear whether such therapy can protect, for example, against extra-articular manifestations such as SSNHL, although biologics and Disease-Modifying Antirheumatic Drugs (DMARDs) focus on systemic inflammation.^25^, ^26^, ^27^, ^28^ Future studies should address whether certain RA therapies are sufficient to pose a threat to SSNHL, what the effect is of the early administration of corticosteroids for idiopathic SSNHL among RA patients, and whether such therapy improves their health as opposed to being administered later on.^29^^,^^30^ This study revealed the need for prompt and interdisciplinary interventions, as rheumatology and otology departments should more effectively be able to handle RA patients with a risk of hearing impairment.

In conclusion, these findings contribute to the expanding evidence explaining that autoimmune diseases like RA can result in SSNHL, likely via an immune-mediated pathway. The finding that RA and SSNHL have a significant relationship that transcends other comorbidities calls for more clinical control measures and approaches for this particular population. More studies need to be conceptualized and tested to define the underlying biological factors that explain how RA is associated with SSNHL and what therapeutic methods can be used to prevent hearing loss from becoming worse.

## Funding

This research was funded by 10.13039/501100010425Tri-Service General Hospital (grant n°: TSGH_E_114295 awarded to Y.-F.W.), The study was supported by grants from Chi-Mei Medical Center (grant n°: 110CM-TMU-16 to K.-H.L. and 111CM-TMU-09 to K.-H.L.), the Higher Education Sprout Project by the Ministry of Education (MOE) in Taiwan (grant n°: DP2-111-21121-01-C-01-03 and DP2-TMU-112-C-05), the Health and Welfare Surcharge of Tobacco Products of Taiwan (Hualien Tzu Chi Hospital Joint Cancer Center; grant n°: MOHW111-TDU-B-221-014013 and MOHW112-TDU-B-221-124013 to K.-H.L.), National Science and Technology Council (grant n°: NSTC 112-2320-B-038-058, NSTC 113-2320-B-038-013, NSTC 113-2320-B-038-042, NSTC 113-2622-B-038-003, and NSTC114-2320-B-038-006 to K.-H.L.), and Taipei Medical University Research Center of Cancer Translational Medicine (Featured Areas Research Center Program, within the framework of the Higher Education Sprout Project by the Taiwanese Ministry of Education).

## Data availability

The data that support the findings of this study are available from the corresponding author upon reasonable request. Access to the data is subject to approval by the National Health Insurance Research Database (NHIRD), Taiwan, due to legal restrictions imposed by the Taiwan Ministry of Health and Welfare.

## Authors’ contributions

Conceptualization: MJC, YFW, and KHL. Data curation: MJC, YCL, and MYC. Formal analysis: CHC and WCC. Funding acquisition: KHL. Investigation: all authors. Methodology: WCC and JHL. Project administration: MJC, YFW, and KHL. Resources: YFW and KHL. Software: YCL, MYC, CHC, and WCC. Supervision: YFW and KHL. Validation: MJC, YCL, and MYC. Visualization: YCL and MYC. Writing-original draft: MJC, YFW, and KHL. Writing-review & editing: all authors. All authors read and agreed to the published version of the manuscript.

## Declaration of competing interest

The authors declare that they have no known competing financial interests or personal relationships that could have appeared to influence the work reported in this paper.

## Appendix Supplementary materials

Image, application 1

## Footnotes

Supplementary material associated with this article can be found, in the online version, at doi:10.1016/j.clinsp.2026.100990.
