# Supplementary material for: Increased incidence of sudden sensorineural hearing loss in patients with rheumatoid arthritis: a nationwide propensity-matched cohort study
Source: Clinics (Sao Paulo). 2026 May 14;81:100990. doi: 10.1016/j.clinsp.2026.100990 (PMC13196373; doi:10.1016/j.clinsp.2026.100990)
Supplement: Supplementary file 1 [file mmc1.doc]

CLINICS-D-25-01037_Supplementary Material

**Supplementary Tables**

**Table S1** Abbreviation, International Classification of Disease 9^th^ Edition Clinical Modification (ICD-9-CM), and definitions.

|  | **Abbreviation** | **ICD‒9‒CM / Definition** |
| --- | --- | --- |
| **Study population:** |  |  |
| Rheumatoid arthritis | RA | 714.0; Outpatient visits ≥ 3 or inpatient |
| **Events:** |  |  |
| Sudden sensorineural hearing loss | SSNHL | 338.2 |
| **Comorbidities:** |  |  |
| Diabetes mellitus | DM | 250 |
| Hypertension | HTN | 401‒405 |
| Depression |  | 296.2‒296.3, 296.82, 300.4, 311 |
| Anxiety |  | 300 excluding 300.4 |
| Chronic kidney disease | CKD | 580‒589 |
| Hyperlipidemia |  | 272 |
| Thyrotoxicosis |  | 242 |
| Septicemia |  | 003.1, 036.1, 038 |
| Pneumonia |  | 480‒486 |
| Chronic liver disease | CLD | 571 |
| Injury |  | 800‒999 |
| Tumor |  | 140‒208 |
| Neurofibromatosis |  | 237.72 |
| **Charlson Comorbidity Index Revised** | CCI_R | CCI removed RA, DM, HTN, CKD, Pneumonia, CLD, and Tumor |

**Table S2‒1** Years of follow-up.

| **RA** | **Min** | **Median** | **Max** | **Mean ± SD** |
| --- | --- | --- | --- | --- |
| With | 0.01 | 7.26 | 15.93 | 9.13 ± 8.65 |
| Without | 0.01 | 7.75 | 15.99 | 9.67 ± 8.94 |
| Total | 0.01 | 7.64 | 15.99 | 9.56 ± 8.89 |

RA, Rheumatoid Arthritis; Min, Minimum; Max, Maximum; SD, Standard Deviation.

**Table S2‒2** Years to sudden sensorineural hearing loss.

| **RA** | **Min** | **Median** | **Max** | **Mean ± SD** |
| --- | --- | --- | --- | --- |
| With | 0.03 | 4.01 | 15.51 | 4.57 ± 3.80 |
| Without | 0.01 | 6.94 | 15.77 | 7.11 ± 4.55 |
| Total | 0.01 | 5.78 | 15.77 | 6.43 ± 4.50 |

RA, Rheumatoid Arthritis; Min, Minimum; Max, Maximum; SD, Standard Deviation.
